# Ist das Delir unabhängig vom Anästhesieverfahren? Was uns REGAIN und RAGA lehren

**DOI:** 10.1007/s00101-022-01104-z

**Published:** 2022-02-23

**Authors:** Josefin Grabert, Mark Coburn

**Affiliations:** Klinik für Anästhesiologie und Operative Intensivmedizin, Universitätsklinikum Bonn, Venusberg-Campus 1, 53127 Bonn, Deutschland

## Originalpublikation

Neuman MD, Fegn R, Canors JL et al (2021) Spinal Anesthesia or General Anesthesia for Hip Surgery in Older Adults. N Engl J Med 385(22):2025–2035.

Li T, Li J, Yuan L et al (2022) Effect of Regional vs General Anesthesia on Incidence of Postoperative Delirium in Older Patients Undergoing Hip Fracture Surgery. JAMA 327:50–58.

## Zusammenfassung der Studien.

Das postoperative Delir (POD) als akut einsetzender, fluktuierender Verwirrtheitszustand ist eine der häufigsten Komplikationen nach großen chirurgischen Eingriffen und kann bis zur Hälfte der Patienten nach kardiochirurgischen oder traumatischen Hüftoperationen betreffen [1, 2]. Eine längere intensivstationäre Therapie und Krankenhausverweildauer sowie höhere Morbidität und Letalität sind nur einige der Folgen [3]. Ob und wie das Anästhesieverfahren die Entstehung eines POD beeinflusst, bleibt unklar. Die 2 kürzlich veröffentlichten Studien REGAIN und RAGA untersuchten vergleichend die Inzidenz des POD als sekundären und primären Endpunkt nach traumatischer Hüftchirurgie unter Allgemein- bzw. rückenmarknaher Regionalanästhesie.

## REGAIN-Studie.

Die multizentrische, pragmatische, randomisierte REGAIN-Studie (Regional versus General Anesthesia for Promoting Independence after Hip Fracture) von Neuman et al. vergleicht Anästhesieverfahren bei proximalen Femurfrakturen. Primärer kombinierter Endpunkt nach 60 Tagen waren Tod und die Unfähigkeit, unabhängig (mit oder ohne Gehhilfe) 3 m zu gehen. Sekundäre Endpunkte waren neu ein aufgetretenes Delir und die Krankenhausverweildauer.

An 46 US-amerikanischen und kanadischen Krankenhäusern wurden alle Patienten ≥50 Jahre mit bevorstehender Operation einer proximalen Femurfraktur für die Studie in Betracht gezogen. Ausschlusskriterien waren eingeschränkte Gehfähigkeit, chirurgische und allgemeine Kontraindikationen einer Spinalanästhesie, maligne Hyperthermie und periprothetische Frakturen. Ein präexistentes Delir war kein Ausschlusskriterium. Nach 1:1-Randomisierung erhielten die Patienten entweder eine einmalige Spinalanästhesie (nach Bedarf mit Analgosedierung) oder eine balancierte Allgemeinanästhesie. An den ersten 3 postoperativen Tagen wurde das POD einmal täglich mittels 3D-CAM (3-Minute Diagnostic Interview for Confusion Assessment Method) erfasst.

In die „Intention-to-treat“-Analyse wurden 795 Patienten für die Spinalanästhesie und 805 Patienten für die Allgemeinanästhesie eingeschlossen. Der primäre Zielparameter Tod oder neue Gangunfähigkeit kam in 18,5 % unter Spinalanästhesie und 18 % unter Allgemeinanästhesie vor (*p* = 0,83; RR 1,03, KI 0,84–1,27). Ein neu aufgetretenes POD wurde in 20,5 % (130/633) nach Spinalanästhesie und 19,7 % (124/629; RR 1,04, KI 0,84–1,3) nach Allgemeinanästhesie beobachtet.

Die Autoren schätzen die Spinal- und Allgemeinanästhesie für proximale Femurfrakturen als gleichwertige Verfahren ein. Sie merken kritisch die additive Analgosedierung zur Spinalanästhesie an, begründen dies aber mit dem pragmatischen Studiendesign. Protokollverletzungen traten in 15 % der Spinalanästhesiegruppe auf, zeigen nach Instrumentenvariablenschätzung aber keinen Unterschied.

## RAGA-Studie.

Die RAGA-Studie von Li et al. untersucht randomisiert, multizentrisch und pragmatisch den Einfluss des Narkoseverfahrens auf die Entwicklung eines POD (primärer Endpunkt) nach proximalen Femurfrakturen. Eingeschlossen wurden an 9 chinesischen Krankenhäusern alle Patienten ≥65 Jahre, bei denen eine chirurgische Frakturversorgung indiziert war. Ausschlusskriterien waren multiple Frakturen, Kontraindikationen für Regional- oder Allgemeinanästhesie, maligne Hyperthermie oder Einschluss in andere Studien.

Präoperativ wurde der Status mittels CAM und MMSE (Mini Mental Status Examination) beurteilt, an den ersten 7 postoperativen Tagen wurde einmal täglich der CAM durchgeführt. Zugelassene Regionalverfahren waren alleinige Spinal‑, Peridural- und kombinierte Spinal- und Epiduralanästhesie. Die Allgemeinanästhesie konnte total intravenös oder balanciert sein. In beiden Gruppen waren Benzodiazepine und Ketamin unzulässig und ein peripherer Nervenblock empfohlen.

In die Intention-to-treat-Analyse eingeschlossen wurden 471 Patienten/Gruppe. Ein neues POD lag in 6,2 % unter Regionalanästhesie und in 5,1 % unter Allgemeinanästhesie vor (*p* = 0,57; RR 1,2; KI 0,7–2,0). Die Schwere des Delirs, gemessen mit der Delirium Rating Scale-Revised-98, und Letalität waren gleich in beiden Gruppen.

Die Autoren betonen die insgesamt niedrige Delirrate unabhängig vom Anästhesieverfahren und begründen dies u. a. damit, dass postoperative Maßnahmen (u. a. Atem‑, Physiotherapie) häufig von Familienmitgliedern durchgeführt werden.

## Kommentar zu den Studien

Das postoperative Delir gewinnt im Rahmen der zunehmenden geriatrischen Chirurgie an Bedeutung. Bekannte prädisponierende Faktoren sind Notfalleingriffe und Medikamente; wie sich das Anästhesieverfahren auf das POD auswirkt, ist bisher jedoch nicht gut untersucht. Beide vorliegenden Studien vergleichen an großen Populationen den Einfluss von Regional- im Vergleich zu Allgemeinanästhesie.

Sowohl in RAGA als auch in REGAIN machte das Anästhesieverfahren für die Delirrate keinen Unterschied. Wenn der Verzicht auf eine zentral wirkende Medikation im Rahmen einer rückenmarknahen Regionalanästhesie das Delir nicht verhindert – lässt sich dann im Umkehrschluss behaupten, dass die Allgemeinanästhesie nicht der ausschlaggebende Faktor in der Entstehung des Delirs ist? Vermeintlich ja. Eine ältere Metaanalyse aus überwiegend retrospektiven Untersuchungen an heterogenen Kollektiven sowie eine zweite Analyse prospektiv gewonnener Daten an älteren Patienten mit hüftnaher Fraktur unterstützen die These [4, 5].

An REGAIN ist anzumerken, dass in der Regionalanästhesiegruppe fast jeder Patient mindestens ein Hypnotikum oder Opioid (44 % Midazolam, 88 % Propofol, 23 % Ketamin) erhalten hat. Zusätzlich wurden 15 % der Regionalanästhesiegruppe in Allgemeinanästhesie operiert, was in einer Intention-to-treat-Analyse nicht berücksichtigt wird. Mit der Gabe prodelirogener Medikamente, die als wichtige prädisponierende Faktoren gelten, lässt sich der fehlende, aber erwartete Unterschied in der Delirrate, nachvollziehen. Umso interessanter ist es, dass in RAGA, wo in der Regionalanästhesiegruppe strikt auf jede zusätzliche Analgosedierung verzichtet wurde, auch in der „Per-protocol“-Analyse kein Unterschied detektiert wurde. Dies steht im Widerspruch zu bisherigen Studien, in denen ein Zusammenhang zwischen tiefer Sedierung und Delirrate gezeigt wurde [6–8]. Denkbar ist, dass nicht die Sedierungstiefe per se, sondern die damit einhergehenden kardiovaskulären Veränderungen (arterielle Hypotonie, Bradykardie, reduziertes Herzzeitvolumen) das Delir begünstigen. Auch dazu ist die Studienlage nicht eindeutig [9–11].

Hervorzuheben an RAGA ist die postoperative Schmerztherapie. Diese ist nicht näher beschrieben, außer dass ca. 20 % der Allgemeinanästhesiegruppe ein peripheres Regionalverfahren erhalten haben. In beiden Gruppen fällt auf, dass kein Patient postoperativ Schmerzen hatte (maximaler Score mittels visueller Analogskala jeweils 0) . Auffallend ist auch die niedrige Delirrate: 6 und 5,1 % in RAGA im Vergleich zu 20 und 19,7 % in REGAIN. Die adäquate Analgesie sowie reorientierende Maßnahmen (frühes und kontinuierliches Einbeziehen von Familienmitgliedern in die Therapie) in RAGA mögen zur niedrigen Delirinzidenz beigetragen haben. Gleichzeitig sind der Anteil an Patienten mit vorbestehendem Delir (13,3 % vs. 0,8 %) und ASA-III-Status (60 % vs. 20 %) in REGAIN deutlich höher. Die Delirinzidenz in REGAIN ist verhältnismäßig niedrig im Vergleich zur Literatur, die mit 5–50 % bei orthopädischen und 10–40 % bei hospitalisierten Patienten angegeben wird [2, 12]. Die in diesen Metaanalysen einbezogenen Studien sind teils älter als 20 Jahre. Vermutlich trägt die insgesamt weiterentwickelte Patientenversorgung, aber auch eine zunehmend spezialisierte und individualisierte Narkoseführung zur Abnahme der Inzidenz bei. Es bleibt abzuwarten, bis weitere große multizentrische Studien, wie z. B. iHOPE abgeschlossen sind. Die Studienprotokolle von REGAIN und iHOPE sind harmonisiert und ermöglichen daher eine Metaanalyse auf Patientenebene (IPD-Metaanalyse) [13].

Was lernen wir aus REGAIN und RAGA? Alle hier eingeschlossenen Patienten sind Hochrisikopatienten für ein Delir: Sie sind alt, sie werden nichtelektiv hüftnah operiert, erhalten einen Blasenkatheter, werden räumlich und zeitlich aus ihrem Umfeld gerissen. Sind die Umstände unabhängig vom Anästhesieverfahren bereits ausreichend für die Entstehung des Delirs? Und warum werden die einen delirant und die anderen nicht? Diese Fragen können RAGA und REGAIN nicht beantworten. Beide zeigen, dass das Anästhesieverfahren nicht der ausschlaggebende Faktor ist. Wahrscheinlich ist es wichtiger, das gewählte Anästhesieverfahren qualitativ hochwertig durchzuführen, als die Verfahrenswahl selbst, und die nichtpharmakologischen Ansätze zu beherzigen [6].

## Fazit für die Praxis

Mit RAGA und REGAIN zeigen 2 große, prospektive und randomisierte kontrollierte Studien, dass eine rückenmarknahe Regionalanästhesie zur Versorgung proximaler Femurfrakturen die Delirinzidenz nicht senkt. Für den klinischen Alltag scheint eine auf den Patienten zentrierte und gut geführte Anästhesie relevanter zu sein als das gewählte Verfahren selbst.
